# Paradoxical indeed

**DOI:** 10.1073/pnas.2504512122

**Published:** 2025-05-05

**Authors:** Zachary T. Compton, Orsolya Vincze, Walker Mellon, Marc Tollis, Lisa Abegglen, Joshua D. Schiffman, Mathieu Giraudeau, Amy M. Boddy, Carlo C. Maley

**Affiliations:** ^a^Arizona Cancer Evolution Center, The Biodesign Institute, Arizona State University, Tempe, AZ 85281; ^b^University of Arizona Cancer Center, Tucson, AZ 85724; ^c^University of Arizona College of Medicine, Tucson, AZ 85724; ^d^Institute of Aquatic Ecology, Centre for Ecological Research, Debrecen 1113, Hungary; ^e^LIENSs, UMR 7266 CNRS-La Rochelle Université, La Rochelle 17000, France; ^f^School of Informatics, Computing and Cyber Systems, Northern Arizona University, Flagstaff, AZ 86001; ^g^Department of Pediatrics and Huntsman Cancer Institute, University of Utah, Salt Lake City, UT 84112; ^h^University of California Santa Barbara, Santa Barbara, CA 93106; ^i^Biodesign Center for Biocomputing, Security and Society, Arizona State University, Tempe, AZ 85724; ^j^School of Life Sciences, Arizona State University, Tempe, AZ 85724

By rejecting Peto’s paradox, Butler et al. demonstrate an unfortunate misunderstanding of the paradox as originally described by Sir Richard Peto (1). Rather than specifying a “lack of association between body size and malignancy risk,” Peto proposed a simple mathematical prediction: An organism’s risk of developing cancer should scale with its total number of somatic cells (i.e., body size) and/or the duration it maintains them (i.e., lifespan). In Peto’s original example, humans have 1,000 times the number of cells than mice and also live 30 times longer, yet humans do not suffer many orders of magnitude more cancer than mice. The implication of this paradox is that larger, long-lived animals must have evolved anticancer defenses to suppress the extraordinary amount of cancer they are expected to develop. Butler et al. report a modest positive correlation between cancer risk and body size across vertebrates, simultaneously claiming to disprove Peto’s paradox while acknowledging that the evolution of enhanced cellular growth control allows species to grow larger “without experiencing the anticipated cancer burden associated with their size” (2). Therefore, the strong assertions made by the authors in the article as well as in the popular press about a lack of evidence for Peto’s paradox is, indeed, paradoxical.

Butler et al. claim “no association has been previously found between cancer prevalence and body size across species”; which overlooks significant evidence from the literature (3–6). These previous studies reveal a modest increase in neoplasms and cancers with increases in body size across taxa. Butler et al. also contend that they found no evidence to suggest exceptionally low malignancy prevalence in Asian elephants, “despite often being touted as the quintessential example of Peto’s paradox.” Rather, several studies have documented that Asian elephants (*Elephas maximus*) are susceptible to both neoplasms [40.9%(4) to 41.4%(5)] and malignancies [0.4%(4) to 14.6%(5)]. Therefore, Butler et al.’s conclusions stem from misinterpreting both theory and literature.

Further, we challenge the evolutionary assumption underlying Butler et al.’s analysis. They use a phylogenetic tree scaled by the rate of body size evolution to test whether enhanced cancer defenses coevolved with a greater rate of body size evolution. However, the *rate* of body size evolution is distinct from the total amount of body size evolution that occurred in a lineage. Rapid body size evolution could lead to evolutionary lag before cancer suppression mechanisms evolve—contrary to their hypothesis. Also, the rate of body size evolution is a nondirectional measure. Lineages that evolved from large to small rapidly would still show high rates of body size evolution. In such cases, smaller descendants may inherit robust cancer defenses from their larger ancestors, with an expected evolutionary lag before these defenses diminish due to shifting evolutionary pressures. Butler et al. mix the two types of body size change, making it difficult to interpret their results.

While we welcome the growing interest in comparative oncology, as an emerging field, we urge caution in making absolute claims given data limitations.
